# Long-Range Rhombohedral-Stacked Graphene through Shear

**DOI:** 10.1021/acs.nanolett.0c01146

**Published:** 2020-06-11

**Authors:** Jean Paul Nery, Matteo Calandra, Francesco Mauri

**Affiliations:** † Graphene Laboratories, Fondazione Istituto Italiano di Tecnologia, Via Morego, I-16163 Genova, Italy; ‡ Dipartimento di Fisica, Università di Roma La Sapienza, Piazzale Aldo Moro 5, I-00185 Roma, Italy; § Sorbonne Université, CNRS, Institut des Nanosciences de Paris, UMR7588, F-75252 Paris, France; ∥ Department of Physics, University of Trento, Via Sommarive 14, 38123 Povo, Italy

**Keywords:** graphene, rhombohedral, long-range ABC order, shear stress, Bernal, density functional theory, friction

## Abstract

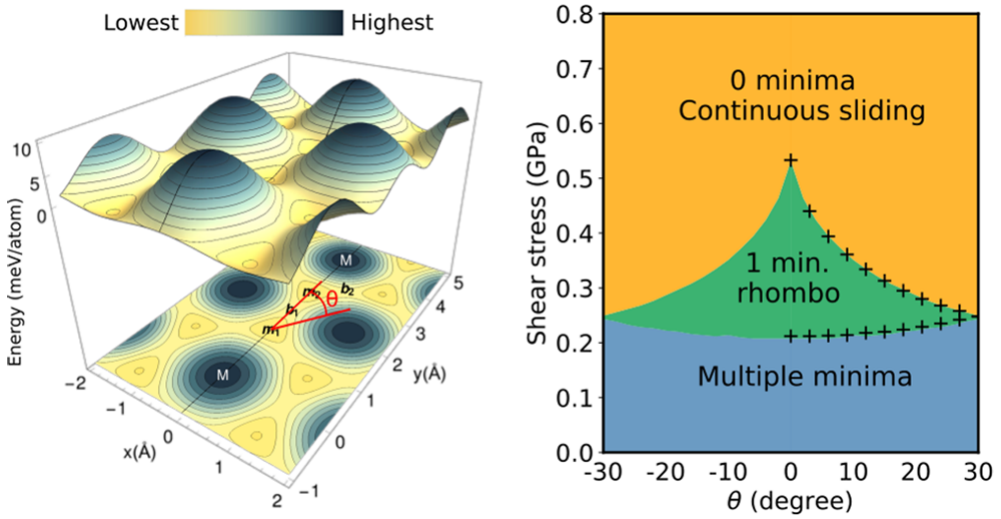

The discovery of superconductivity and correlated electronic states in the flat bands of twisted bilayer graphene has raised a lot of excitement. Flat bands also occur in multilayer graphene flakes that present rhombohedral (ABC) stacking order on many consecutive layers. Although Bernal-stacked (AB) graphene is more stable, long-range ABC-ordered flakes involving up to 50 layers have been surprisingly observed in natural samples. Here, we present a microscopic atomistic model, based on first-principles density functional theory calculations, that demonstrates how shear stress can produce long-range ABC order. A stress-angle phase diagram shows under which conditions ABC-stacked graphene can be obtained, providing an experimental guide for its synthesis.

Multilayer graphene exhibits two main types of stacking. In Bernal-stacked multilayer graphene (BG), layers are stacked repeatedly in the AB sequence, while in rhombohedral-stacked multilayer graphene (RG), the stacking is ABC. The main interest in RG stems from its flat bands close to the Fermi energy,^1,2^ which could lead to exciting phenomena such as superconductivity,^3−6^ charge-density wave, or magnetic orders.^7^ The extent of the flat surface band in the Brillouin zone and the number of electrons hosted increases with the number of consecutive ABC-stacked layers (saturating at approximately 8 layers).^2,8,9^ Thus, mastering the thickness of ABC flakes is a way to tailor correlation effects. However, RG is much less common than the energetically favored BG phase^10^ and does not appear isolated.^11−13^ While superconductivity has already been measured in twisted bilayer graphene,^14−16^ work on RG has been slower because of the inability to consistently grow or isolate large single crystal samples.

X-ray diffraction experiments^10,17^ have shown that some natural samples contain small amounts of rhombohedral graphite. However, such experiments have not determined if the stacking is random, or if there are many consecutive layers of ABC-stacked graphene, namely, if there is a phase separation between BG and RG. With these same limitations, also using X-ray diffraction, it was qualitatively noticed that shear strain increases the percentage of rhombohedral inclusions in Bernal graphite.^10^ Ref (18) proposed a gliding mechanism, but it involved going through an intermediate AA stacking, a high-energy state. Then, ref (10) proposed gliding that avoided AA stacking and involved a shorter displacement. This pinpointed the gliding mechanism that produces RG but did not explain the precise nature of the stacking.

Definitive experimental evidence of long-range ABC order has only been obtained in the past several years. After applying shear to BG, over 10 consecutive layers of RG were first observed using high-resolution transmission electron microscopy.^19^ More than 14 layers^20,21^ and up to 50 layers of RG^22,23^ have been observed in exfoliated samples as well. Notice that, for a random stacking, the probability of obtaining *N* consecutive layers of RG is 1/2^*N*–2^, which corresponds to only 0.02% for *N* = 14 and becomes extremely small for *N* ∼ 50. Thus, there must be some underlying reason, either energetic or kinetic, that explains why this happens.

Here, we propose a mechanism to produce long-range RG staking from BG using shear stress. In particular, we use an atomistic model, based on first-principles calculations, to obtain a stress-angle phase diagram that identifies the conditions for the formation of RG. The required stress is similar to that already realized in friction experiments of graphene.^24,25^

## Mechanical Model

**Figure 1:**
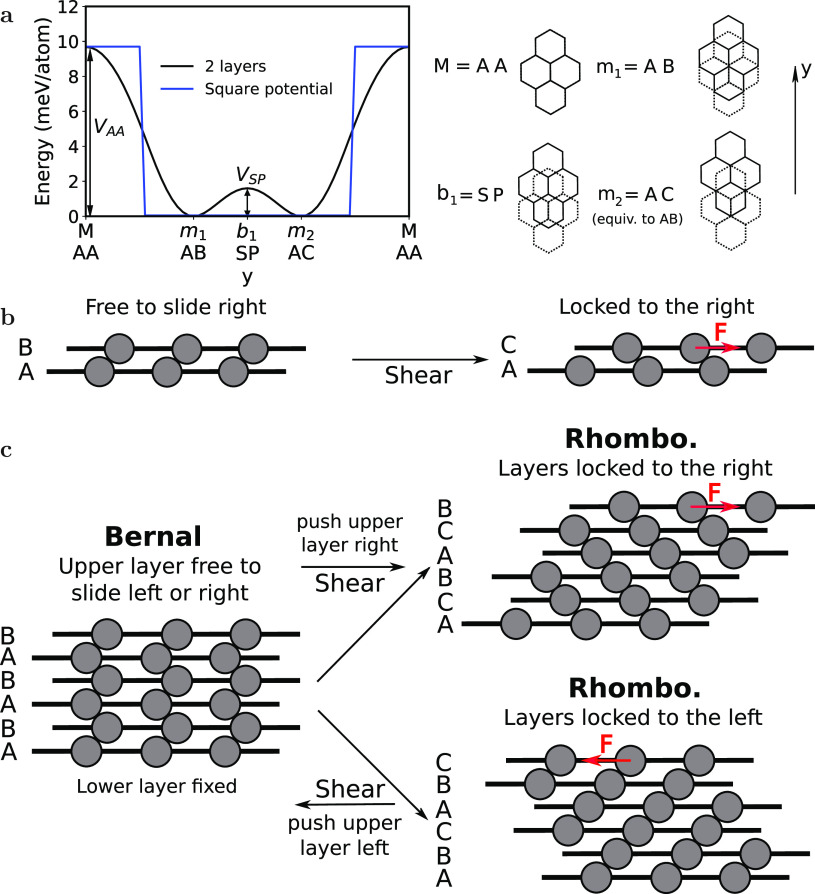
Transformation from BG to RG in mechanical model. (a) Black: one dimensional (1D) interaction energy per interface atom (see the DFT calculations section in the Supporting Information), which we refer to as potential, of a two-layer graphene system, where the upper layer moves with respect to the lower one along the armchair direction. Energies at *m*_1_ = AB and *m*_2_ = AC are the same and have the lowest energy. AA stacking (M) is the least favorable configuration, with energy *V*_AA_. *b*_1_ = SP is a barrier, with energy *V*_SP_, that the upper layer needs to overcome to go from one minima to the other. Blue: simplified square version of the black potential, where the lower barrier *V*_SP_ is set to 0. The height going to infinity (hard wall) corresponds to the mechanical model in parts b and c. (b) 1D mechanical model. The dark circles can be thought of as hard carbon atoms. Letters on the left label the position of each layer. On the left, the upper layer can move without resistance to the right. After the upper layer is pushed to the right, it gets “locked”. We refer to this as a sliding step. (c) Analogous to part b, but considering multiple layers. The lower layer is fixed in position A. The top layer is pushed to the right (upper right diagram). After successive sliding steps, all layers end up locked in the ACBACB configuration. Similarly, when pushing the upper layer to the left, layers end up locked in ABCABC.

A simple mechanical model is used to explain the underlying mechanism in the transformation of multilayer BG to RG via shear stress. We start by considering the interaction energy of two layers of graphene, in which the upper layer moves relative to the lower one, fixed in what is referred to as position A. Calculations are carried out within density functional theory (DFT)^26−30^ with an LDA functional, since parameters like the shear frequency agree well with experiment (see the DFT calculations section in the Supporting Information for details on why LDA is a good functional for our purposes). A layer of graphene on top of another one (configuration AA) corresponds to a maximum of interaction energy. The most stable configuration is obtained when one of the graphene atoms of the upper layer is right above an atom of the lower layer, and the other atom is equidistant from six carbon atoms in the lower layer. There are two of these configurations, AB and AC, both corresponding to a Bernal bilayer. See Figure 1a. In configuration SP, the upper layer is in the middle of positions B and C. It corresponds to a saddle point in the full 2D bilayer energy, which we refer to as potential *V*. Throughout the whole paper, we consider the energy per interface atom (see the DFT calculations section the Supporting Information). The full energy curve, when moving the upper layer relative to the lower layer along the bond direction (also known as the armchair direction), starting from AA, results in the black curve of Figure 1a.

The mechanical model corresponds to a simplified version of the 1D potential of Figure 1a: the low barrier around SP separating the two minima is neglected (flat region of width 2*d*), while the high barrier around AA is considered as a hard wall (infinite potential of width *d*). It corresponds to the square potential in Figure 1a when the height goes to infinity. Then, each layer, considering the interaction with an upper and lower layer, can be regarded just as a rigid block of width *d* connected by rods of size 2*d*. To make the visualization easier, we consider circles instead of blocks and assume that each layer can only move horizontally. The circles can be thought of as hard carbon atoms. In Figure 1b, the upper layer is free to move to the right, until it makes contact with the lower layer, getting “locked”. This happens repeatedly when considering several layers: the movement of the upper layer translates into stress on the lower layers as well (if the transformation is quasistatic, the stress on each layer is the same as the one on the top), resulting in long-range rhombohedral order. If shear is applied as in the top part of Figure 1c, by exerting a force on the upper layer, ABABAB transforms into ACBACB (if the force is applied in the opposite direction, it transforms into ABCABC). That is, BG transforms into RG (a more detailed description is included in the Mechanical model section of the Supporting Information). Experimentally, stress on the upper layer can be achieved, for example, by using a cantilever, the tip of an STM,^31^ or in encapsulation, by movement of the layer attached to the upper graphene layer (like hexagonal boron nitride^22^).

## Transformation from BG to RG: First-Principles Calculations

**Figure 2:**
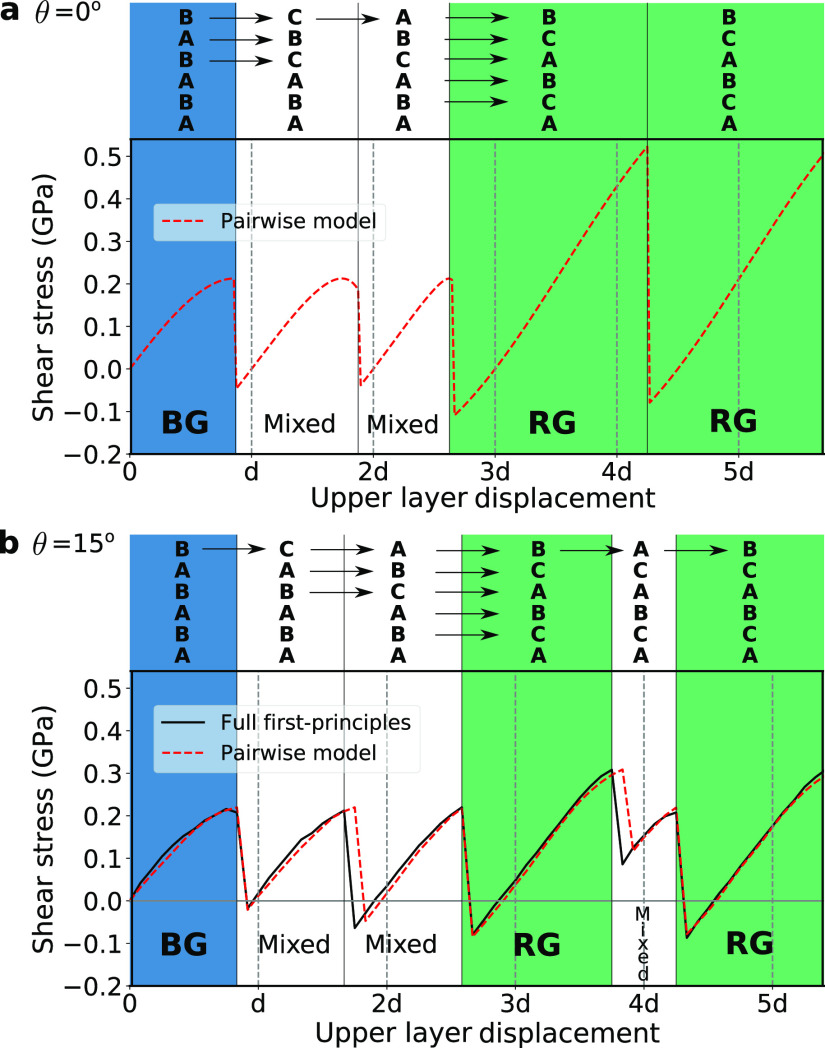
Transformation from BG to RG in first-principles calculation. (a) Shear stress along the armchair direction *y* (θ = 0°) of a 6-layer calculation, as a function of the center of mass displacement of the upper layer. Only the interaction between nearest layers is considered. BG is transformed into RG by applying shear stress on the upper layer. The lower layer is fixed, and the upper layer is moved and fixed in steps of *d*/12 (with *d* the bond length) in *y* (the perpendicular direction is relaxed). All other coordinates are relaxed. The letters on the top of each region indicate the stacking sequence into which the system relaxes to when the external stress is removed. The arrows indicate which layers change position. The initial configuration all in blue is BG, while the configuration all in green is RG. As the upper layer moves in +*y*, it is pushed in −*y* toward the original equilibrium configuration BG. The external stress increases until a sliding step takes place at the critical stress of about 0.2 GPa, and the stress decreases abruptly or “jumps”. After 3 sliding steps, RG is formed, and layers are “locked” in +*y*. Now the stress increases until about 0.5 GPa (big critical stress); the upper layer moves in the perpendicular direction *x* (see Figure S3a), but the structure remains fully rhombohedral. (b) Analogous to part a, but with θ = 15°. The black curve is a full first-principles calculation. The excellent agreement between this pairwise model and the full first-principles calculation shows that the pairwise model is sufficient to study transformations when shear is applied. RG is also formed after 3 sliding steps. Then, stress increases to about 0.3 GPa; the upper layer jumps in *x*, but now the system is not fully rhombohedral. After a sliding step, however, RG is recovered, and the sequence continues repeating itself. Notice how the small critical stress does not depend much on the angle, whereas the big critical stress is significantly smaller for θ = 15°.

Here, we consider an analogous transformation to that of the mechanical model of the previous section, using two calculations. In one case, we consider only the interaction between nearest layers, in what we refer to as the pairwise model. The other is a full DFT-LDA calculation. The calculations agree very well (see Figure 2b), showing that the pairwise potential is sufficient to study how the layering sequence changes with shear stress. The main qualitative difference with the one-dimensional mechanical model of the previous section is that, after layers are locked in RG, they move in the perpendicular direction if the external stress increases too much.

**Figure 3:**
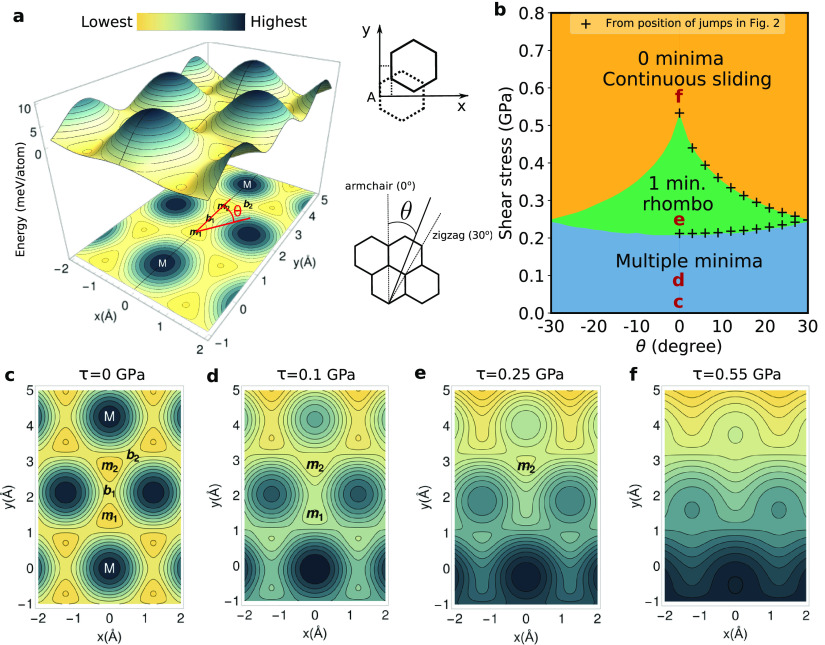
Bilayer potential phase diagram. (a) Energy per interface atom *V* (DFT fit) of a bilayer graphene system, with the center of mass of the upper layer moving relative to the lower layer. The coordinate system is indicated on the top right. Energy is higher in darker regions and lower in lighter regions, as indicated in the color bar. The lower layer is fixed in position A, and a projection on the plane is also displayed below. The angle θ, illustrated also on the bottom right diagram, indicates the direction of the shear stress **τ** with respect to the armchair direction. The slice *x* = 0 is shown in black, just as in Figure 1a. (b) Stress-angle phase diagram obtained by counting the number of minima of the enthalpy *H*(**r**) = *V*(**r**) – **τ**·**r***A*, with *A* the area of the flake. (i) Blue region: 2 minima (multiple minima in an *N* layer system). System remains in the current local minima, which is BG if that is the starting point (the most stable structure when there is no stress). (ii) Green region: 1 minimum. The relative position of a layer relative to the lower one is always the same, so the phase is RG. (iii) Orange region: there is no local minima, so the system slides continuously. The plus signs “+” are obtained from calculations as in Figure 2 and correspond to the critical values of stress before it decreases abruptly. For each angle, the small critical stress gives the lower plus sign and the big critical stress the upper one. We see they agree very well with the borders between the regions determined from the minima analysis (lower crosses match the blue-green border and upper crosses the green-orange border). Thus, the minima analysis is sufficient to characterize the system. A transition to RG may occur at lower values due to thermal fluctuations. Contour plots at the positions of the letters c–f at θ = 0° are shown below. (c) Same as contour plot in part a. There are two equivalent minima *m*_1_ and *m*_2_, separated by barriers *b*_1_ and *b*_2_. (d–f) *H* as a function of *x* and *y* for values of stress corresponding to the blue, green and orange regions, with 2, 1, and 0 minima, respectively.

The center of mass of the lower layer is fixed in all calculations (it can be thought of as attached to a substrate like copper or nickel, that have a larger shear stress), and the upper layer is “pushed” by a fraction of the bond length *d* along the direction that makes an angle θ with the armchair direction (see Figure 3a). In each step, the structure is relaxed (more details in the Transformation calculations section of the Supporting Information). Figure 2 shows the external force per unit area (shear stress) on the center of mass of the upper layer, for θ = 0° (a) and θ = 15° (b) (the component of the force perpendicular to the θ component is 0, since the upper layer is relaxed in that direction). We consider a quasistatic transformation, so the external force is minus the force exerted by the rest of the system.

The initial configuration is ABABAB (six layers of BG, in blue). Let us first consider θ = 0°. As the upper layer starts to move in +*y* from its initial position B, the rest of the system tries to restore it to the equilibrium position BG, and stress increases. When it reaches the critical stress of about 0.2 GPa (which we refer to as small critical stress), it drops abruptly, and the system moves toward the nearest minimum, ABABAC. A sliding step has taken place, analogous to Figure 1b. In the literature, this type of gradual movement followed by sudden jumps (see also Figure S3) is known as “stick–slip” motion.^24,32^ The regions are delimited by the points where the force drops abruptly and are labeled on top by the structure the system relaxes to upon releasing the external force. The arrows indicate the layers that are changing position from one region to the next one. After the first sliding step, two additional sliding steps take place, and RG (green) is formed; thus, all layers are locked. The stress increases now until a larger critical stress of around 0.5 GPa (big critical stress); the upper layer suddenly changes *x*_CM_ (Figure S3a), moving “around” the maxima that the high-stress configuration is close to, and the force decreases significantly. In this case, rhombohedral order remains. For θ = 15°, after 3 sliding steps RG is also formed, but after the big critical stress rhombohedral order is partially lost. However, after one sliding step RG is obtained again. The small critical stress is around 0.2 GPa, similar to that of θ = 0°, but the big critical stress is around 0.3 GPa, which differs considerably from 0.5 GPa. There is indeed a significant angle dependence for the big critical stress, as can be observed later in more detail in Figure 3b.

Thus, if the magnitude of the applied stress is lower than the small critical stress, around 0.2 GPa, the layering will not change. It will stay as BG after removing the stress. If stress is between the small and big critical stresses, the final structure will be RG. If the applied stress is larger than these limiting upper values, layers will keep on sliding.

Additionally, it is important to point out that if the lower layer is fixed in position A, there is only one possible BG stacking AB, while there are two possible RG stackings ABC and ACB. The RG stackings are related by a 180° rotation. There are two inequivalent armchair directions: while θ = 0° leads to ACB stacking, θ = 180° leads to ABC stacking. This is analogous to the two possible transformations in Figure 1. If the stacking is ACB, at θ = 0° layers are locked and RG is preserved, while θ = 180° gradually destroys rhombohedral order. Experimentally, it is of interest to transfer RG to a substrate (like in encapsulation in ref (22)), which involves shear stress. Thus, to keep the rhombohedral order, the destroying armchair direction should be avoided. See Stability of RG in the Supporting Information for more details.

## Full 2D Bilayer Potential

The excellent agreement between the pairwise model and the first-principles calculations (Figure 2b) suggests it should be possible to characterize an *N* layer system in terms of the building block of the pairwise model, the potential *V*, shown in Figure 3a. We will now show this is indeed the case. *V* was obtained by considering the upper layer in different positions with respect to the lower one (see details in the Fourier Interpolation section of the Supporting Information). The potential along the *x* = 0 line, shown in black, is the same as in Figure 1a. The top right of Figure 3a indicates the system of coordinates: the lower layer (dashed) is fixed and determines the origin, while the center of mass position of the upper layer determines the *x*, *y* coordinates.

## Phase Diagram

As mentioned earlier, depending on the magnitude and angle of the stress applied, the system can be BG, RG, or slides continuously. When the system is subjected to a shear stress **τ** = **F**/*A*, where **F** is the applied force and *A* the area of the flake, it can be studied using the bilayer enthalpy *H*(**r**) = *V*(**r**) – **τ**·**r***A*. As we will now see, the number of minima of *H* determines the phase the system is in, giving a stress-angle phase diagram (Figure 3b) for multilayer graphene.

In the pairwise model, if a stress **τ** is applied to the upper layer in a quasistatic transformation, then the layer below exerts all the remaining stress −**τ**. The same applies to subsequent layers. Thus, for each pair of layers, their enthalpy *H* is the same. Depending on the angle and magnitude of **τ**, there are three possible situations, which correspond to the 3 colored regions of Figure 3b:

(i) Blue region: 2 minima. If no stress is applied, *H* = *V*, and there are 2 minima *m*_1_ and *m*_2_ (see Figure 3c). If shear stress is sufficiently low, *H* still has 2 minima, and the system has 2^*N*–1^ minima. For each pair of layers, they do not escape the local minimum they are currently in. Since this holds for all layers, the full system does not escape the local minimum it is currently in. In particular, if BG is chosen as the starting structure, the system remains BG in the blue region (in the pairwise model, all stacking sequences have the same energy, but in reality BG is the most stable structure).
(ii) Green region: 1 minimum. As stress increases, *m*_1_ disappears, and *m*_2_ remains close to its **τ** = 0 position. Barrier *b*_1_ disappears, while *b*_2_ remains. This occurs because the stress is more aligned with the direction in which the saddle point *b*_1_ has a maximum (that is, the direction in which *b*_1_ acts as a barrier) than with the corresponding direction of *b*_2_ (except at θ = 30°, where both barriers are affected in the same way, and the system transitions directly from 2 minima to 0 minima). Since there is only one minimum, all layers are in the same position relative to the lower layer, and the resulting stacking is rhombohedral. This is a key observation of our work. From the convention in Figure 1a, *m*_2_ corresponds to configuration AC, and layers are ACB-stacked. If stress is applied in the opposite direction, *m*_1_ is the only remaining minimum, and layers are ABC-stacked.
(iii) Orange region: 0 minima. For larger stresses, there are 0 minima. Since there are no local minima, layers keep on sliding without reaching a stable configuration. If the stress is eventually removed, the system will not necessarily be RG. However, transformations at different angles as in Figure 2 suggest that the system will still have a high degree of rhombohedral order.

In particular, for small angles, the structure remains fully RG. Also, θ = 0° is the angle with the largest range of stress that results in RG, of about 0.3 GPa (from 0.2 to 0.5 GPa). Thus, the armchair direction is the most robust direction to obtain RG.

Figure 3b also shows with plus signs “+” the critical stress values. For each angle, the small critical stress corresponds to the lower value, and the big critical stress to the upper value. They coincide with the blue-green border, and green-orange border, respectively. This excellent agreement shows that the number of minima of *H* does indeed define the stress-angle phase diagram.

It is worth pointing out that when *m*_1_ becomes shallow enough, it might be possible for thermal fluctuations to excite layers from *m*_1_ from *m*_2_. This will depend on experimental conditions, like duration of the experiment, temperature, and size of the flakes. Thus, the curve that separates multiple minima from 1 minimum is actually an upper bound. Also, BG is more stable than RG and is presumably located in a deeper (local) minima (in an analogous fashion to the two minima of the blue curve of Figure S4). Thus, for θ close to 30°, it might occur that the system transitions directly from BG to continuous sliding.

## Pressure

Other hydrostatic pressures were also considered. Figure S8 shows the phase boundaries at *P* = 0 (blue-green and green-orange borders of Figure 3b, or lower and upper borders) and *P* = 2 GPa. The values of the lower and upper boundary at θ = 0° increase approximately linearly with pressure, at about 0.07 and 0.18 GPa of shear stress per GPa of hydrostatic pressure, respectively. Thus, the amount of stress needed to obtain RG increases, but also the range of allowed values to obtain RG (which might increase the robustness of an experiment).

## Shear in Previous Works

The values of stress to produce RG suggested by our calculations are very similar to values already published in experimental reports. The configurations we have described, where layers are commensurate with each other, are referred to as *lock-in* states in the graphene literature related to friction or shear. In this type of system, values of shear stress of the order of 0.1 GPa were measured^24^ and observed to be in good agreement with previous calculations.^32^ In another experiment,^25^ a microtip of a micromanipulator was used to apply a shear force on a graphene flake to “unlock” it (remove it from the minima), and based on the deformation of the tip, a value of 0.14 GPa was reported, also lower than 0.20 GPa. On the other hand, when the layers are incommensurate with each other, the values of friction are 2 or 3 orders of magnitude lower. This phenomenon is referred to as superlubricity and has sparked a lot of interest. Optimal conditions for superlubricity include big flakes, low temperatures, and low loads.^33^ Since layers have to be moved out of the local minimum in the mechanism we have proposed, depending on the experimental conditions, care might need to be taken to avoid the upper layer to rotate into a superlubricant state.

There are previous experimental works that have produced a few layers of ABC graphene by using chemical vapor deposition and tailoring the curvature of the substrate,^34^ using a perpendicular electric field,^35,36^ doping,^36^ and twisting.^37^ These techniques might provide possible routes to produce long-range RG, but so far it has only been observed in samples involving exfoliation or milling (and thus shear).^19−23^ The advantage of our method is that any single crystal could be in principle transformed into RG, as opposed to searching for RG in exfoliated samples, which is much more time-consuming.

To conclude, we have described a mechanism to transform multilayer graphene into RG through shear stress, which implies that applying sufficient shear strain to graphite results in long-range RG. Also, existing experimental values of shear stress are similar to the ones suggested by our results. Our model suggests a compelling method for experimental groups trying to obtain multiple layers of rhombohedral-stacked graphene.

## Supporting Information Available

DFT calculations, the mechanical model, transformation calculations, stability of RG along different shear directions, Fourier interpolation of the potential, hydrostatic pressures, shear stress as a function of the displacement of the upper layer along the zigzag direction, change of *y*_CM_ and *x*_CM_ of each layer for the calculations of Figure 2, energy of the bilayer system and of a six-layer calculation along the armchair direction, energy of the bilayer system along the armchair direction at a fixed distance and for the rvv10 functional, interlayer distance of the bilayer system along the armchair direction for various hydrostatic pressures, plots analogous to those of Figure 1a at several hydrostatic pressures, phase boundaries at several hydrostatic pressures, shear stress on the upper layer as a function of *y*_CM_ of the upper layer for various numbers of layers, and a table comparison between the bilayer potential and the 6-layer calculation (PDF)
