# Supplementary material for: Long-Range Rhombohedral-Stacked Graphene through Shear
Source: Nano Lett. 2020 Jun 11;20(7):5017–23. doi: 10.1021/acs.nanolett.0c01146 (PMC8008449; doi:10.1021/acs.nanolett.0c01146)
Supplement: Supplementary file 1 — nl0c01146_si_001.pdf [file nl0c01146_si_001.pdf]

Supporting Information to

Long-range Rhombohedral-stacked Graphene Through Shear

Jean Paul Nery[1,2,*], Matteo Calandra[3,4], and Francesco Mauri[1,2]

[1]*Graphene Labs, Fondazione Istituto Italiano di Tecnologia, Via Morego, I-16163 Genova, Italy.*

[2]*Dipartimento di Fisica, Università di Roma La Sapienza, Piazzale Aldo Moro 5, I-00185 Roma, Italy.*

[3]*Sorbonne Université, CNRS, Institut des Nanosciences de Paris, UMR7588, F-75252, Paris, France.*

[4]*Department of Physics, University of Trento, Via Sommarive 14, 38123 Povo, Italy.*

email: nery.jeanpaul@gmail.com

DFT calculations. Calculations were performed in Quantum Espresso (QE)[2] using an LDA functional. The bilayer potential and the six layer first-principles transformations were obtained with a cutoff of 80 Ry, a k-grid of $56 \times 56 \times 1$ and an electronic temperature (Fermi-Dirac smearing) of 284 K. The energies considered in this work are always in meV per interface atom. This means that the total energy of the system is divided by 2, whether the number of layers is 2 or 6 (which permits a direct comparison between energies of both systems). In each system, the energy is set to 0 at the relative position where the energy is the lowest. To converge the energy differences of Table S1 with a precision below 0.01 meV/atom, we used an energy cutoff of 120 Ry and an electronic grid of $80 \times 80 \times 1$. In the rvv10 calculations of Figure S5, 100 Ry were used.

The most important parameter in the calculations is basically the small barrier V_{SP} of Figure 1a between the two minima, since it determines the amount of stress necessary to go

from one local minimum to another. The value of V_{SP} can be expected to be somewhat accurate if the curvature around the minima is accurate. The curvature is directly proportional to the frequency squared of the shear mode LO' at Γ (this is the mode in which layers move in the plane in opposite directions).[3] Bulk experimental values of the shear frequency vary between 42 cm^{-1} and 45 cm^{-1}(ref. 4). This is in good agreement with the value that results from fitting a parabola close to m_1 in Figure 1a and considering nearest neighbors for the bulk value, 42 cm^{-1}. The curvature of other functionals like rvv10 (ref. 5), which includes van der Waals, is actually lower in our calculations, so they agree less well with experiments (see Figure S5). In previous works, rvv10 also gives a lower frequency than LDA.[6]

To be confident that it is sufficient to consider the 2 layer potential in our analysis, we considered a similar calculation, but with 6 layers (dotted-blue line in Figure S4) instead of 2. In each calculation, the x_{CM} and y_{CM} of each of the three upper layers was moved with respect to the three lower layers, and the system was relaxed. This corresponds to a generalized stacking fault energy.[7] The curves are very similar, and the energy difference between the minima is about 6% of V_{SP}, so the transformations could be analyzed in terms of the bilayer potential. Indeed, the curves in Figure 2 are almost identical. Also, the value obtained for the stacking fault energy, 1.58 meV/atom, compares well with 1.53 meV/atom obtained with RPA.[8] A detailed comparison is included in Table S1.

Mechanical model. Let us label Li the ith layer from the bottom to the top, and let us consider that a force is applied to the right on the upper layer L6, as in the top part of Figure 1c. First, L6 moves from B to C without resistance, where it locks with L5. Then, it pushes L5 from A to B, which in turn pushes L4 from B to C. The lower layers have not moved yet. In the next step, L2 and L3 move as well. The transformation is (labeling always from bottom to top): ABABAB→ABABAC→ABACBA→ACBACB. If the direction is reversed, the steps of the transformation are: ABABAB→ABABCA→ABCABC. In both cases, RG is obtained.

Transformation calculations. As stated in the main text, in each step, the upper layer LN is moved in the direction θ by a fraction d/N_{step} of the bond length d (and relaxed in the perpendicular direction). The center of mass of the lower layer is fixed, and the intermediate layers are fully relaxed (the same applies in the Stability of RG section below). Each intermediate layer $i = 2, .., N-1$ is actually moved as well in $[(i-1)/(N-1)]d/N_{\text{step}}$, giving a configuration closer to the equilibrium position. In addition, each intermediate layer is displaced by a relatively large $d/20$ in the perpendicular direction, to smoothen the asymmetry introduced by the displacement of the upper layer. In this way, different layers move in the perpendicular direction at the critical stress, as opposed to only the upper layer (layers are equivalent in our model and small fluctuations in position should change the layer that jumps in different calculations).

A careful treatment is also needed at $\theta = 0°$. There is a bifurcation of behavior at the critical stress: a layer jumps in the perpendicular direction in $-a/2$ for $\theta < 0$, and $a/2$ for $\theta > 0$. So the result of Figure S1 actually corresponds to $\theta = 2°$, to avoid an artificial behavior at $\theta = 0°$ ($\theta = 2°$ can be made smaller by increasing N_{step} and reducing the displacement $d/20$ in the perpendicular direction).

Stability of RG. As pointed out in the main text, the most convenient direction to obtain RG is an armchair direction (there are two inequivalent ones, $\theta = 0°$ and $\theta = 180°$, that give ACB and ABC stacked RG, respectively). After RG is formed, or if is obtained for example from exfoliated flakes like in ref. 1, it is of interest to know how stable it is when applying shear along different directions (which occurs, for example, when transfering it to a substrate).

To study this, we use the pairwise model and 20 layers of ACB-RG as a starting structure. The upper layer is moved a fixed total amount of about $10d$ (14.4 Å) along different θ. We define a rhombohedral fraction R_s (see details below), which determines the rhombohedral character of a flake. It is higher when its absolute value is closer to 1: it is 1 when the stacking is ACB, -1 when it is ABC. Other configurations, like random stacking or BG, are

closer to 0.

Calculations are performed as in the previous section, with the only difference that the upper layer is displaced in smaller steps of $d/24$. As mentioned in the main text, there are two inequivalent armchair directions, which we refer to as preserving ($\theta = 0°$) and destroying ($\theta = 60°$) directions. If the starting structure is ABC instead of ACB stacked, the preserving and destroying directions are reversed. Results can be seen in Figure S1. $\theta = 0°$ corresponds to the curve after $3d$ in Figure 2a, for which the rhombohedral order is kept ($R_s = 1$). The opposite direction, $\theta = 180°$ (equivalent to $\theta = 60°$), corresponds to the reverse transformation, in which rhombohedral order is gradually lost (R_s decreases instead of increasing).

For $0° \leq \theta < 30°$, the stacking sequence remains rhombohedral or one sliding step away, resulting in $R_s > 0.9$. At $\theta = 30°$ (zigzag) there is a discontinuity, since $R_s = 0.94$ at $\theta = 29°$, while $R_s = 0.52$ at $\theta = 31°$. For $31° \leq \theta \leq 60°$, R_s varies between 0.50 and 0.52. Surprisingly, rhombohedral order is gradually destroyed for any angle in this range, just as for $\theta = 60°$.

Thus, if starting from BG, there are two reasons of why an armchair direction is the most convenient direction to apply shear stress: (i) it has the largest difference between the small and large critical stress, making it easier to avoid the continuos sliding region of the phase diagram; (ii) in the stability analysis, R_s has the highest value. If in an experiment the starting structure is RG, the appropriate armchair direction would have to be determined.

In ref. 1, it is observed that the armchair direction destroys rhombohedral order. However, a distinction between the two type of armchair directions is not made, and stress could have been applied in the destroying direction. Also, it is not known what is the magnitude of the applied shear stress, or the precision of the transfer angle. If there are sign fluctuations in the transfer process, the armchair direction would destroy RG, while the zigzag direction might preserve it, since 30° and −30° are equivalent ($\theta = 30°$ is in the middle of $\theta = 0°$ and $\theta = 60°$, which create and destroy ACB order, respectively, and could intuitively be useful to

preserve the existing stacking sequence as mentioned in ref. 1). With regards to our model, a more detailed analysis that takes into account temperature and dynamical effects could give a smoother behavior around the zigzag direction. However, this is beyond the scope of our work.

Stability of RG: Rhombohedral fraction. For a given stacking sequence, let us assign +1 or -1 to the interface between consecutive layers in the following way: +1 for layers in positions AC, CB, BA and -1 for CA, BC, AB. For example for $N = 7$, ACBACBA $\rightarrow$ +1+1+1+1+1+1, while ABABABA $\rightarrow$ -1+1-1+1-1+1. Adding these values and dividing by $N-1$ gives R_s. So for ACBACBA, $R_s = 1$, and for ABABABA, $R_s = 0$. In general, $R_s = 1$ for ACB stacking, and $R_s = 0$ for AB stacking if N is odd (if N is even, $R_s = -1/(N-1)$ for AB stacking, $1/(N-1)$ for BA stacking, the closest possible values to 0). Equivalently, R_s can be defined as $R_s = 1 - 2n_s/(N-1)$, where n_s is the number of layers that can slide in $+y$ (with a small critical stress) relative to the layer below.

To obtain Figure S1, every time the force drops, the structure is relaxed and R_s is determined. Then R_s is averaged. This procedure is repeated 50 times for each angle, and a final averaged R_s is calculated.

Fourier interpolation. To obtain the bilayer potential at an arbitrary $\mathbf{r}$ point as in Figure 3a (see coordinate system there), and to then obtain the phase diagram, the potential was first calculated at a set of N discrete points $\mathbf{r}_i = (x_{\mathrm{CM},i}, y_{\mathrm{CM},i})$ of the upper layer within a primitive cell (separated by $a/15$ along the lattice vectors, with $a = 2.46$ Å the length of the lattice vectors, totaling $N = 225$). As mentioned earlier, the interlayer distance and relative coordinates were relaxed. Let $\mathbf{G}$ be a set of N reciprocal lattice vectors around the origin with the symmetry of the crystal. Then, we can write

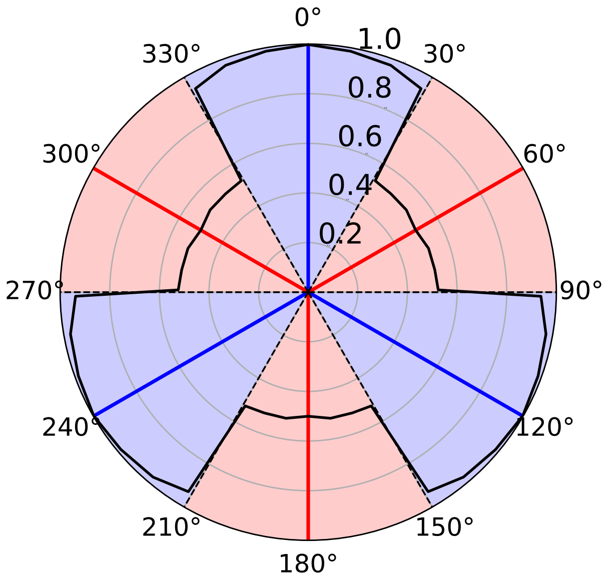

Figure S1: Rhombohedral fraction R_s as a function of the angle of the applied stress, starting from $N = 20$ layers of ACB-RG, which has $R_s = 1$. BG has $R_s = 0$ and ABC-RG $R_s = 1$. When applying shear to RG, some of the rhombohedral order can be lost (layers are not locked anymore). There are two armchair directions, $\theta = 0°$ in blue and $\theta = 60°$ in red, with $120°$ periodicity. Zigzag directions ($\theta = 30°, 90°, ..., 330°$) are all equivalent. θ around $0°$ is the most stable direction, with $R_s = 1.00$. For $0° < \theta < 30°$, the structure is RG or one sliding step away. At the zigag direction there is a discontinuity, and rhombohedral order gets gradually destroyed at $30° < \theta \leq 60°$. The other angles can be determined from symmetry considerations. Angles in the blue areas preserve rhombohedral order to a high degree, while angles in the red areas destroy it.

$$\begin{aligned} V(\mathbf{r}) &= \sum_{\mathbf{G}} V_{\mathbf{G}} e^{i\mathbf{G}.\mathbf{r}} \\ \text{with } V_{\mathbf{G}} &= \frac{1}{N} \sum_{\mathbf{r_i}} V(\mathbf{r_i}) e^{-i\mathbf{G}.\mathbf{r_i}} \end{aligned} \tag{1}$$

where $\mathbf{r}$ can take any value.

Hydrostatic pressure. In order to obtain a phase diagram as in Figure 3b when there is an external pressure P, we have to add an additional term to the enthalpy H defined earlier, resulting in the new enthalpy

$$H(\mathbf{r}) = V(\mathbf{r}) - \boldsymbol{\tau} \cdot \mathbf{r} A + PzA, \tag{2}$$

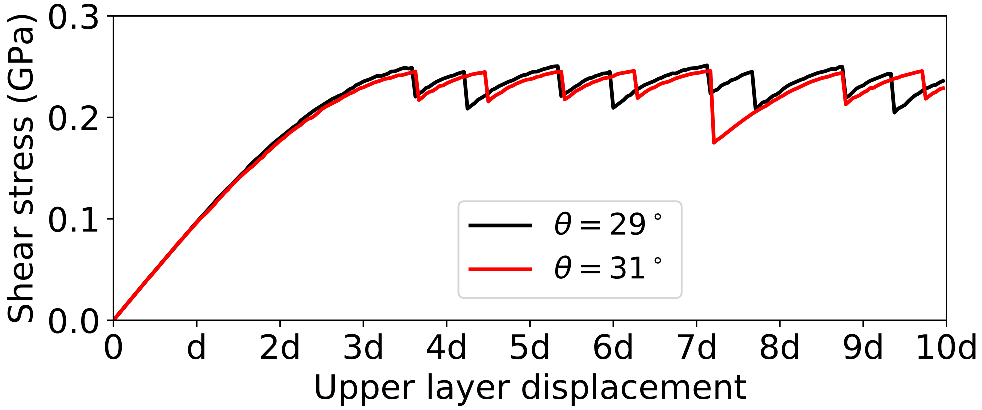

Figure S2: Shear stress as a function of the displacement of the upper layer for a system of 20 layers. The starting configuration is RG, and the angles are 29° (black) and 31° (red). For $\theta = 29^\circ$, there are two type of peaks, with slighlty different heights. When the structure relaxes to RG, the peak is slightly higher (large critical stress), and it is lower when it does not (small critical stress). In the latter case, the structure is only 1 sliding step away from RG. In Figure 2, for $\theta = 0^\circ$ and $\theta = 15^\circ$, these values differ more, while all peaks have the same height at $\theta = 30^\circ$ (barriers b_1 and b_2 are equivalent). For $\theta = 31^\circ$, all peaks correspond to the low critical stress and have the same height, as the lower value of the black curve. At the positions where the black and red peaks overlap, it can be seen that the red peak is slightly lower. Rhombohedral order is gradually destroyed, just as it happens for $\theta = 60^\circ$. The critical values here, of around 0.25 GPa, can also be observed in the phase diagram of Figure 3.

where z is the interlayer distance of the center of mass at pressure P.

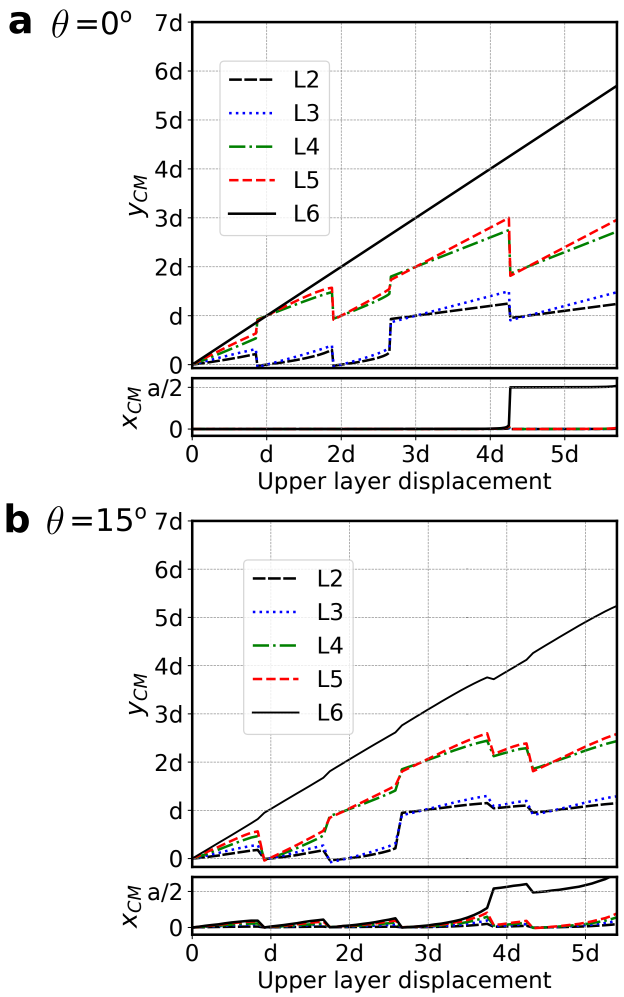

Figure S3: **a**, Change of $y_{\rm CM}$ and $x_{\rm CM}$ of each layer, as a function of the center of mass position $y_{\rm CM}$ of the upper layer (L6), for the 6 layer pairwise model calculation (see Figure 2a). Starting from ABABAB, the upper layer is "pushed" along the bond direction: 1/12 of the bond length d is added to $y_{\rm CM}$ of the upper layer in each step, and then the structure is relaxed. The relaxed coordinates are used in the subsequent calculation. In the first sliding steps, layers only move in y. After RG is formed, stress increases until the upper layers jumps in x in $a/2$. **b**, Analogous plot, with $\theta = 15°$. In this case, the structure does not remain fully rhombohedral after the jump in x, but is soon recovered after a sliding step in y.

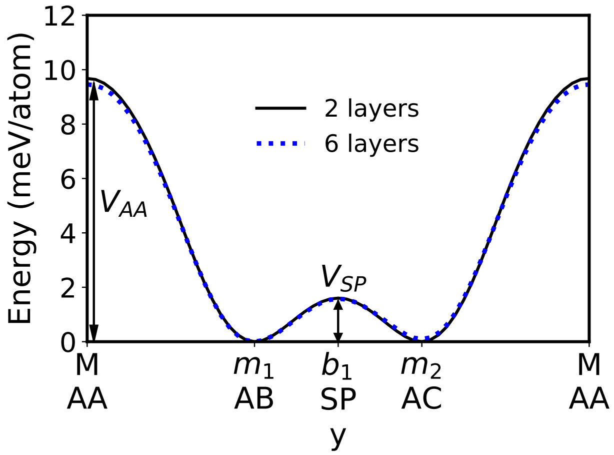

Figure S4: Full-black: Energy of the bilayer system along the armchair direction. Same as in Figure 1a. Dotted-blue: Similar calculation with 6 as opposed to 2 layers. The center of mass of the lower layers is fixed in the configuration ABA in all calculations, while the upper 3 layers move (the center of mass of each layer is fixed in each calculation, while other coordinates are relaxed). Labels m_1 and m_2 correspond to ABABAB and ABACBC, respectively. The energy difference corresponds to a stacking fault energy. M corresponds to ABAACA. The similarity between the curves indicates that the 2 layer potential is a good approximation to analyze how shear affects the stacking order. See Table S1 for a more detailed comparison.

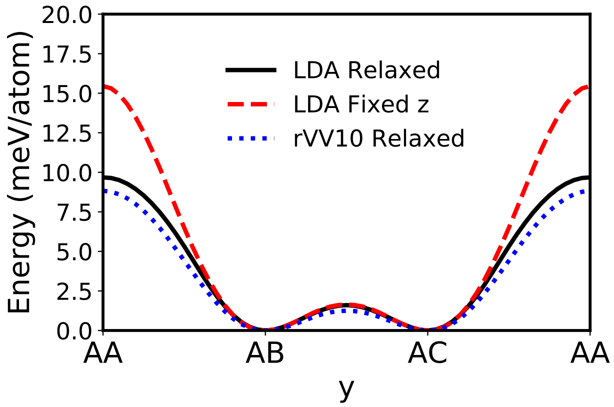

Figure S5: Energy of the bilayer system along the armchair direction. Full-black: LDA relaxed calculation (same as Figure 1a). Dashed-red: Fixed z (at the relaxed value of the AB configuration). Dotted-blue: Relaxed rvv10. Although in the AB-AC region the agreement is good, the disagreement between both curves increases as the configuration approaches AA. This is consistent with Figure S6, which shows the variation of the interlayer distance $z_{\rm CM}$. $z_{\rm CM}$ is 3.38 Å at the small barrier, close to 3.33 Å at AB, while at AA is 3.60 Å. Thus, it is important to relax the configuration. Otherwise, the potential differences are artificially high.

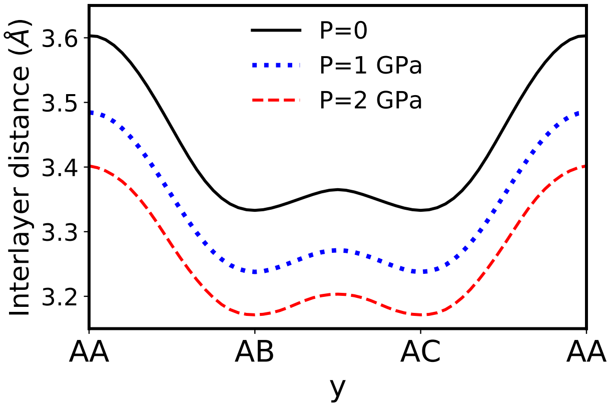

Figure S6: Interlayer distance of the bilayer system along the armchair direction, for various hydrostatic pressures. Full-black: $P = 0$. Dotted-blue: $P = 1$ GPa. Dashed-red: $P = 2$ GPa.

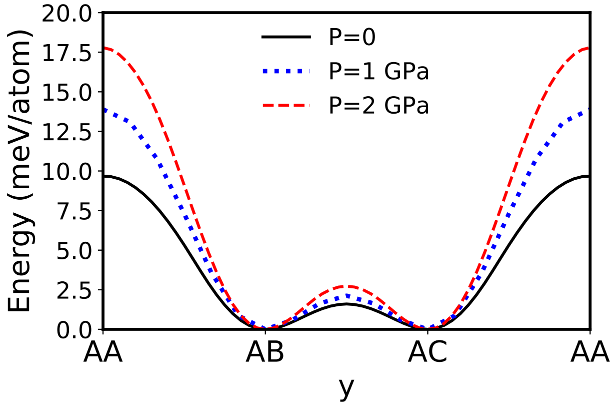

Figure S7: Plots analogous to those of Figure S4 at varying pressures. Full-black: $P = 0$ (same as Figure 4). Dotted-blue: $P = 1$ GPa. Dashed-red: $P = 2$ GPa. Figure S5 shows that fixed z close to AA corresponds to artificial pressures of over 1 GPa.

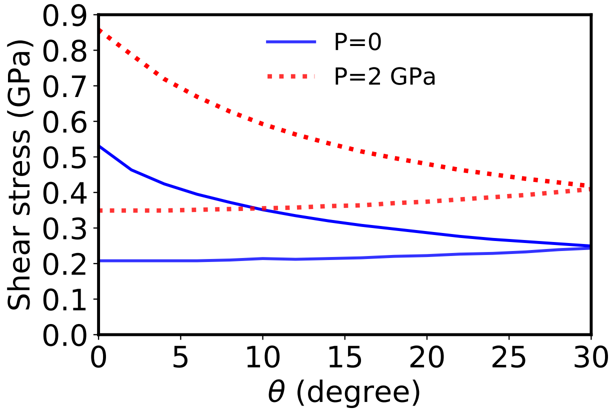

Figure S8: Phase boundaries at different hydrostatic pressures. For each color, the lower line corresponds to the border between the blue and green region of the phase diagram (Figure 3b), and the upper curve to the border between the green and orange regions. Blue: $P = 0$ (same as in Figure 3b). Red: $P = 2$ GPa. In this range of pressures, the values of the curves at $\theta = 0^\circ$ increase linearly with pressure.

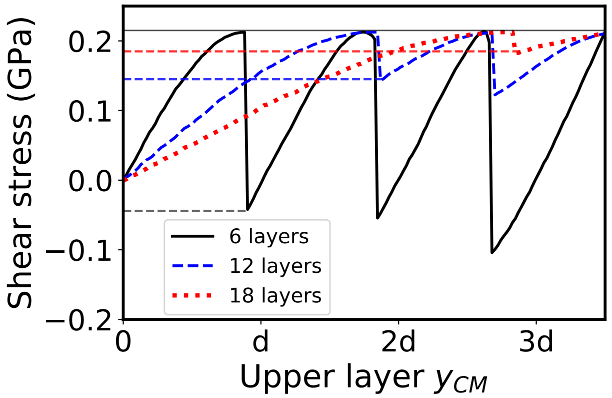

Figure S9: Shear stress on the upper layer as a function of y_{CM} of the upper layer. The jump of stress, as sliding steps take place, decreases with the number of layers. The jump is also smaller after RG is formed. This can be useful to apply a less varying and more controllable stress. If for example a cantilever is used to move the upper layer, the number of layers of the sample would have to be taken into account to decide on its optimal elastic constant. It can also be seen how the position of the first jump increases linearly with the number of layers.

Table S1: Comparison between the 2 layer potential and the 6 layer calculation of Figure S4, and previous works. Refs. 7 and 8 use the adiabatic-connection fluctuation-dissipation theorem within the random phase approximation (ACFDT-RPA). Ref. 9 uses anisotropic elasticity theory, revised values for the elastic constants and the experimental data of refs. 10 and 11, to obtain an average stacking fault energy of 0.14 meV per interface atom, the same value as the RPA calculation. The difference between the minima (stacking fault energy) is small compared to the barrier V_{SP} separating them, which implies the 2 layer potential provides a good approximation to study the transformation through shear.

	Energy (meV/atom)		
	2 layer potential	6 layer potential	Previous works
Stacking fault Bernal	0	0.10	0.14 (RPA)[7]<br>0.14 (Exp.)[9]
Small barrier V_{SP}	1.58	1.59	1.53 (RPA)[8]
Large barrier V_{AA}	9.7	9.5	8.8 (RPA)[8]<br>12.4 (QMC)[12]

References

1. Y. Yang, Y.-C. Zou, C. R. Woods, Y. Shi, J. Yin, S. Xu, S. Ozdemir, T. Taniguchi, K. Watanabe, A. K. Geim, K. S. Novoselov, S. J. Haigh, and A. Mishchenko, Stacking Order in Graphite Films Controlled by van der Waals Technology, Nano Lett. **19**, 8526-8532 (2019).

2. P. Giannozzi, S. Baroni, N. Bonini, M. Calandra, R. Car, C. Cavazzoni, D. Ceresoli, G. L. Chiarotti, M. Cococcioni, I. Dabo, A. Dal Corso, S. Fabris, G. Fratesi, S. de Gironcoli, R. Gebauer, U. Gerstmann, C. Gougoussis, A. Kokalj, M. Lazzeri, L. Martin-Samos, N. Marzari, F. Mauri, R. Mazzarello, S. Paolini, A. Pasquarello, L. Paulatto, C. Sbraccia, S. Scandolo, G. Sclauzero, A. P. Seitsonen, A. Smogunov, P. Umari, R. M. Wentzcovitch, J.Phys.:Condens.Matter **21**, 395502 (2009); P. Giannozzi, O. Andreussi, T. Brumme, O. Bunau, M. Buongiorno Nardelli, M. Calandra, R. Car, C. Cavazzoni, D. Ceresoli, M. Cococcioni, N. Colonna, I. Carnimeo, A. Dal Corso, S. de Gironcoli, P. Delugas, R. A. DiStasio Jr, A. Ferretti, A. Floris, G. Fratesi, G. Fugallo, R. Gebauer, U. Gerstmann, F. Giustino, T. Gorni, J. Jia, M. Kawamura, H.-Y. Ko, A. Kokalj, E. Küçükbenli, M. Lazzeri, M. Marsili, N. Marzari, F. Mauri, N. L. Nguyen, H.-V. Nguyen, A. Otero-de-la-Roza, L. Paulatto, S. Poncé, D. Rocca, R. Sabatini, B. Santra, M. Schlipf, A. P. Seitsonen, A. Smogunov, I. Timrov, T. Thonhauser, P. Umari, N. Vast, X. Wu. and S. Baroni, J.Phys.:Condens.Matter **29**, 465901 (2017).

3. N. Mounet and N. Marzari, First-principles determination of the structural, vibrational and thermodynamic properties of diamond, graphite, and derivatives, Phys. Rev. B **71**, 205214 (2005).

4. I. V. Lebedeva, A. V. Lebedev, A. M. Popov, and A. A. Knizhnik, Comparison of performance of van der Waals-corrected exchange-correlation functionals for interlayer interaction in graphene and hexagonal boron nitride, Computacional Material Science **128**, 45-58 (2017).

5. R. Sabatini, T. Gorni, and S. de Gironcoli, Nonlocal van der Waals density functional made simple and efficient, Phys. Rev B **87**, 041108(R) (2013).

6. R. R. Del Grande, M. G Menezes and, R. B. Capaz, Layer breathing and shear modes in multilayer graphene: a DFT-vdW study, J. Phys.: Condens. Matter **31**, 295301 (2019).

7. W. Wang, S. Dai, X. Li, J. Yang, D. J. Srolovitz, and Q. Zheng, Measurement of the cleavage energy of graphite, Nat. Comm. **6**, 7853 (2015).

8. S. Zhou, J. Han, S. Dai, J. Sun, D.J. Srolovitz, Van der Waals bilayer energetics: Generalized stacking-fault energy of graphene, boron nitride, and graphene boron nitride bilayers, Phys. Rev. B **92**, 155438 (2015).

9. R. H. Telling and M. I. Heggie, Stacking fault and dislocation glide on the basal plane of graphite, Philos. Mag. Lett. **83**, 411421 (2003).

10. C. Baker, Y. T. Chou, and A. Kelly, Phil. Mag. **6**, 1305 (1961).

11. S. Amelinckx, P. Delavignette, and M. Heerschap, Chem. Phys. Carbon **1**, 1 (1965).

12. E. Mostaani, N. D. Drummond, V. I. Falko, Quantum Monte Carlo calculation of the binding energy of bilayer graphene, Phys. Rev. Lett. **115**, 115501 (2015).
